# Hormone replacement therapy enhances IGF-1 signaling in skeletal muscle by diminishing miR-182 and miR-223 expressions: a study on postmenopausal monozygotic twin pairs

**DOI:** 10.1111/acel.12245

**Published:** 2014-07-18

**Authors:** Fabiola Olivieri, Maarit Ahtiainen, Raffaella Lazzarini, Eija Pöllänen, Miriam Capri, Maria Lorenzi, Gianluca Fulgenzi, Maria C Albertini, Stefano Salvioli, Markku J Alen, Urho M Kujala, Giulia Borghetti, Lucia Babini, Jaakko Kaprio, Sarianna Sipilä, Claudio Franceschi, Vuokko Kovanen, Antonio D Procopio

**Affiliations:** 1 Department of Clinical and Molecular Sciences, Division of Pathology, Università Politecnica delle MarcheAncona, Italy; 2 Department of Clinical Pathology and Innovative Therapy, Advanced Technology Center for Aging Research, INRCA-IRCCSAncona, Italy; 3 Department of Health Sciences, University of JyväskyläJyväskylä, Finland; 4 Gerontology Research Center, University of JyväskyläJyväskylä, Finland; 5 Department of Experimental Pathology, University of BolognaBologna, Italy; 6 Department of Experimental and Clinical Medicine, Division of Neuroscience and Cell Biology, School of Medicine, Università Politecnica delle MarcheAncona, Italy; 7 Dipartimento di Scienze Biomolecolari, Sezione di Biochimica e Biologia molecolare, Università degli Studi di Urbino “Carlo Bo”Urbino, Italy; 8 Department of Medical Rehabilitation, Oulu University Hospital and Institute of Health Sciences, University of OuluOulu, Finland; 9 Department of Public Health, University of HelsinkiHelsinki, Finland; 10 Institute for Molecular Medicine, University of HelsinkiHelsinki, Finland; 11 National Institute for Health and WelfareHelsinki, Finland

**Keywords:** aging, AKT, FOXO3A, IGF-1 signaling, IGF-1R, menopause, miR-142-3p, miR-182, miR-223, mTOR, phosphorylation

## Abstract

MiRNAs are fine-tuning modifiers of skeletal muscle regulation, but knowledge of their hormonal control is lacking. We used a co-twin case–control study design, that is, monozygotic postmenopausal twin pairs discordant for estrogen-based hormone replacement therapy (HRT) to explore estrogen-dependent skeletal muscle regulation via miRNAs. MiRNA profiles were determined from *vastus lateralis* muscle of nine healthy 54–62-years-old monozygotic female twin pairs discordant for HRT (median 7 years). MCF-7 cells, human myoblast cultures and mouse muscle experiments were used to confirm estrogen’s causal role on the expression of specific miRNAs, their target mRNAs and proteins and finally the activation of related signaling pathway. Of the 230 miRNAs expressed at detectable levels in muscle samples, qPCR confirmed significantly lower miR-182, miR-223 and miR-142-3p expressions in HRT using than in their nonusing co-twins. Insulin/IGF-1 signaling emerged one common pathway targeted by these miRNAs. IGF-1R and FOXO3A mRNA and protein were more abundantly expressed in muscle samples of HRT users than nonusers. *In vitro* assays confirmed effective targeting of miR-182 and miR-223 on *IGF-1R* and *FOXO3A* mRNA as well as a dose-dependent miR-182 and miR-223 down-regulations concomitantly with up-regulation of FOXO3A and IGF-1R expression. Novel finding is the postmenopausal HRT-reduced miRs-182, miR-223 and miR-142-3p expression in female skeletal muscle. The observed miRNA-mediated enhancement of the target genes’ *IGF-1R* and *FOXO3A* expression as well as the activation of insulin/IGF-1 pathway signaling via phosphorylation of AKT and mTOR is an important mechanism for positive estrogen impact on skeletal muscle of postmenopausal women.

## Introduction

Menopause results from the loss of ovarian function and is defined as a natural cessation of menstruation that usually occurs after the fifth decade of a woman’s life. Consequently, women spend more than one-third of their lives in postmenopausal status. The menopausal decline in estrogen can have wide-ranging effects on women’s health, as it contributes to decreased bone mass and density (Riggs *et al*., 2002), to the accumulation and redistribution of adipose tissue (Tchernof *et al*., 2004) and to increased risk for metabolic disorders and cardiovascular diseases (Carr, 2003). Furthermore, growing evidence indicates that age-related changes in hormonal status play a role in the pathogenesis of sarcopenia (Kamel *et al*., 2002). A low level of muscle mass and strength increases the risk for type II diabetes and may lead to functional disability and loss of autonomy and finally to premature death. To prevent and treat sarcopenia effectively, the background molecular mechanisms need to be known.

The role of estrogen during growth and development is clear: in interaction with other hormones, it is essential for bone and muscle development where it delivers its effects via specific receptors. However, the role of estrogen in aging muscle is not as well understood as it is in bone. A recent meta-analysis, nevertheless, concluded that as a result of improved muscle quality rather than muscle hypertrophy, postmenopausal women using hormone replacement therapy (HRT) had ~5% greater muscle strength than those not using HRT (Greising *et al*., 2009). It has been suggested that a low postmenopausal circulating estrogen level with increased pro-inflammatory cytokines is an indirect mechanism in the progression of sarcopenia (Roubenoff, 2003). Furthermore, estrogen can also have a direct effect on muscle metabolism, as estrogen receptors, along with the enzymes needed for local estrogen synthesis, are expressed in skeletal muscle (Lemoine *et al*., 2003; Wiik *et al*., 2003; Pöllänen *et al*., 2011). Our previous studies have shown that HRT has favorable effects on lean body mass, muscle mass and performance during early postmenopausal years (Sipilä *et al*., 2001; Taaffe *et al*., 2005) and that long-term HRT use is associated with better mobility, greater muscle power and favorable body and muscle composition (Ronkainen *et al*., 2009). Some of the benefits of HRT may be implemented through the muscular IGF-1-pathway, the activity of which we have recently observed to be higher in HRT users than nonusers (Pöllänen *et al*., 2010; Ahtiainen *et al*., 2012a). The IGF-1-pathway plays a central role in muscle mass regulation, as it activates protein synthesis and inhibits protein degradation (Velloso, 2008). Therefore, modulation of the IGF system is potential strategy to improve muscle condition in older adults.

Recently, a class of evolutionary conserved small noncoding RNAs, termed microRNAs (miRNAs), has been characterized (Lim *et al*., 2005). MiRNAs bind to the 3′UTR of target mRNAs and either block the translation of the message or bind to the open reading frame and target the mRNA transcript for degradation. The actions of miRNAs are sequence- or motif-specific but not gene-specific. Consequently, the dysregulation of miRNA expression can lead to alterations in the expression of hundreds of mRNAs and proteins. During the past few years, the regulation of miRNAs by estrogen has been confirmed, not in healthy human tissue but in breast cancer tissue and/or breast cancer cells (Le Quesne & Caldas, 2010). In normal mouse cells, estradiol has been shown to modulate miRNA expression (Dai *et al*., 2008). With respect to skeletal muscle, recent findings support the hypothesis that key aspects of muscle biology are subject to regulation by muscle-specific myomiRNAs (van Rooij *et al*., 2009; Carvajal & Rigby, 2010). It is highly likely that hormones play an important role in regulating the expression of miRNAs in muscle and therefore in regulating of muscle properties. However, to date, no previous studies have attempted to identify estrogen-responsive miRNAs in skeletal muscle or any other healthy human tissue. The identification and characterization of estrogen-regulated miRNAs can be expected to provide new biomarkers and therapeutic targets in the aging body as well as in aging-related disorders, including sarcopenia.

In the present study, we sought to identify estrogen-regulated miRNAs and their mRNA/protein targets in the skeletal muscle tissue of nine healthy, postmenopausal monozygotic (MZ) female twin pairs discordant for HRT. This design enables us to study the differences between HRT users and nonusers independently of interindividual sequence-level genetic variability. In addition to genetic factors, co-twins also share a number of environmental factors, starting from their intrauterine environment and continuing throughout childhood. The results from human studies were finally confirmed *in vitro* with human muscle cell experiments and by using mouse mature muscle cells in culture.

## Results

### Participant characteristics

Table 1 presents the participants’ body composition and hormonal status according to use of HRT. The mean age of the participants was 57.8 ± 2 years. As expected, the concentration of 17β-estradiol (E_2_) was on average five times higher in the HRT users than in their nonusing co-twins (*P* = 0.003). Body fat percentage was smaller in the HRT users than nonusers (*P* = 0.031). The relative muscle area in the cross section of the thigh was larger (*P* = 0.009), and concomitantly, the relative fat area was smaller (*P* = 0.009) in the HRT users than nonusers. In vertical jumping test, 20% greater muscle power was observed in the HRT users than in nonusers (*P* = 0.012). Furthermore, the participants did not differ regarding diseases, physical activity, smoking behavior, alcohol consumption, or daily energy intake expressed as% amount of energy obtained from proteins, fat, or carbohydrates (Ronkainen *et al*., 2009). Additionally, according to the questionnaires administrated for the Finnish Twin Cohort in 1975, 1981, and 1990 (Kaprio & Koskenvuo, 2002), there were no differences in physical activity, smoking behavior, or alcohol use between these HRT users and their nonusing co-twins.

**Table 1:** Basic characteristics of postmenopausal genetically identical twin sister pairs from whom the other twin was a current HRT user (7.5 ± 3.9 years), and the co-twin had never used HRT. Data are expressed as means ± SD. *P*-values are obtained from paired-samples *t*-test

Variables	Nonuser *n* = 9	HRT user *n* = 9	*P*
Estradiol (pmol L^−1^)	35.3 ± 29.7	190.9 ± 220.5	0.003
BMI (kg m^−2^)	26.5 ± 5.0	24.7 ± 3.5	0.153
Percentage fat (%)	33.3 ± 7.9	28.8 ± 6.4	0.031
Relative muscle area in thigh cross section (%)	52.1 ± 11.0	56.3 ± 8.8	0.009
Relative fat area in thigh cross section (%)	47.9 ± 11.0	43.7 ± 8.8	0.009
Muscle power: vertical jumping height (cm)	13.7 ± 4.4	16.5 ± 2.9	0.012

### MiRNA expression profile comparison in MZ twin pairs discordant for HRT

**Figure 1:**
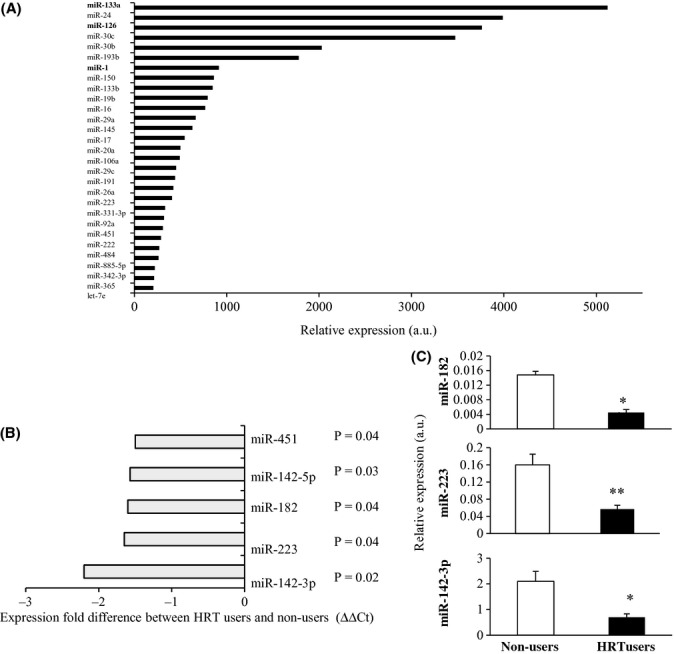
Expression of miRNAs in skeletal muscle of postmenopausal MZ twin pairs. (A) The most highly expressed miRNAs in skeletal muscle of the co-twins not using HRT. Data are normalized against the median relative expression value of all detectable miRNAs and expressed as the relative expression values in arbitrary unit (a.u.). MyomiRNAs are presented in bold. (B) Differentially expressed miRNAs in the Applied Biosystems miRNA Array pool A in muscle samples of HRT using and nonusing MZ co-twins. Each bar corresponds to the expression fold difference, calculated as ΔΔCt, of the miR listed in the figure. (For detailed calculations for the values, see the experimental procedures section). Data are reported as the mean value of three independent experiments. A 1.5-fold or greater difference with a *P*-value < 0.05 (paired-samples *t*-test) was considered significant and reported in the figure. (C) Differentially expressed miRNAs in skeletal muscle of nonusers (white bar) and HRT users (black bar) as measured by qPCR. Data were normalized against *RNU44* expression levels. *P* values are from paired-samples *t*-test. *P ≤ 0.05, **P ≤ 0.01.

To identify differentially expressed miRNAs in the skeletal muscle biopsy, samples were screened for the expression of 365 miRNAs using microarray assay. MiRNAs expressed at a detectable level in more than 80% of samples were included, and using this filtering criterion, 230 miRNAs were taken into the final analysis. The most highly expressed miRNAs in the HRT nonusers, representing postmenopausal muscle at baseline, are shown in Fig. 1A. MiR-133a, one of the specific myomiRNAs, proved to be the most abundant miRNA in skeletal muscle, with an expression rate about 5000-fold greater than the median of all the miRNAs expressed. Of the 230 miRNAs expressed at detectable levels, only five showed higher than 1.5-fold changes (*P* < 0.05). MiR array data showed miR-142-3p, miR-142-5p, miR-223, miR-182, and miR-451 to be hypo-expressed in the HRT users compared to nonusers (Fig. 1B). Validation by quantitative PCR (qPCR) confirmed that the expression levels of miR-182, miR-223, and miR-142-3p in the HRT users were approximately one-third of that of nonusers (*P* = 0.05, 0.001 and 0.003, respectively; Fig. 1C), while miR-142-5p and miR-451 were not significantly different between HRT users and their nonuser co-twins.

### Identification of putative common mRNAs and pathways targeted by differentially expressed miRNAs

**Figure 2:**
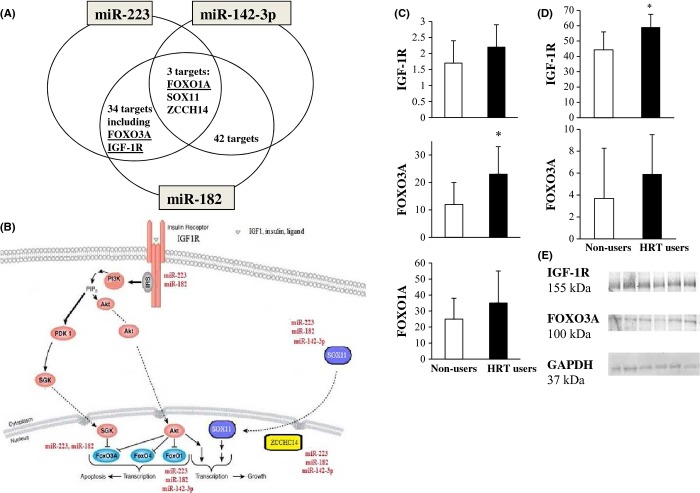
Putative common targets of the identified estrogen-responsive miRNAs and their putative role in the insulin/IGF1 signaling pathway. (A) Venn diagram showing the common target genes of the identified miRNAs. The complete lists of all putative common targets are reported in Table S1. (B) Putative role of the target genes in insulin/IGF-1 signaling pathway. (C) Gene expression of potential common miR-targets with significant role in the insulin/IGF-1 pathway in skeletal muscle of postmenopausal women. Gene expression was measured by qPCR. (D) Western blot analyses of target proteins normalized against GAPDH. Protein levels were analyzed by densitometry and normalized against GAPDH expression. (E) Representative Western blot images from three twin pairs always presenting first the nonuser followed with her HRT-using co-twin. *P* values are from paired-samples *t*-test. *P ≤ 0.05.

To identify putative mRNA target sequences shared by two or three of the identified miRNAs, we used a simple Fortran program combining miRNAs and related target predictions documented in the PicTar and TargetScan databases (SID1.0, Albertini *et al*., 2011). Three putative mRNA targets common to all three identified miRNAs were found: *FOXO1A*, *SOX11,* and *ZCCH14*. In addition, pair-wise analyses revealed 42 common targets for miR-182 and miR-142-3p, 34 common targets for miR-182 and miR-223, and 7 common targets for miR-142-3p and miR-223 (Table S1, Fig. 2A).

Identification of common pathways targeted by miR-182, miR-223, and miR-142-3p was obtained using the DIANA-microT 3.0 target prediction program (http://diana.cslab.ece.ntua.gr/microT/), which has been shown to have the highest ratio of correctly predicted targets over other prediction tools (Maragkakis *et al*., 2009). DIANA-microT 3.0 calculates the miRNA-targeted gene score -log(*P* value), which reflects the weighted sum of the scores of all the conserved and nonconserved miRNA recognition elements on the 3′UTR of the target mRNA, and this score was also indicated for clusters of miRs. This score has been demonstrated to correlate well with fold changes in suppression of protein expression (Maragkakis *et al*., 2009; Satoh & Tabunoki, 2011). Putative pathways affected by miR-182, miR-223, and miR-142-3p are reported in Table 2. Among the putative target pathways, we found the insulin/IGF1 pathway (Fig. 2B). As recently we reported that estrogen-based hormone therapy is associated with increased activity in the same pathway (Pöllänen *et al*., 2010; Ahtiainen *et al*., 2012a), we focused here on *IGF-1R*, *FOXO3A,* and *FOXO1A*, which in our analysis proved to be regulated by miR-182, miR-223, and miR-142-3p, as described in Fig. 2.

**Table 2:** Common pathways of miR-142-3p, miR-182, and miR-223. KEGG Pathway name, the identification ID, and miRNA-targeted gene score (−log (*P*-value) are shown. The database used for this analysis was DIANA-MicroT 3.0

KEGG Pathway name	ID	−log(*P*-value)
Regulation of actin cytoskeleton	hsa04810	17.66
Focal adhesion	hsa04510	8.31
Colorectal cancer	hsa05210	7.62
ErbB signaling pathway	hsa04012	5.76
IGF1/Insulin signaling pathway	hsa04910	4.85

### mRNA and protein levels of putative miRNA targets in muscle tissue of identical twins discordant for HRT

The mRNA levels of the putative miRNA target genes belonging to the insulin/IGF-1 signalling pathway were analyzed by qPCR. The mean transcript levels of *IGF-1R*, *FOXO1A,* and *FOXO3A* in the HRT users were 128%, 123%, and 181% of the mean values of their nonusing co-twins (Fig. 2C). However, only *IGF-1R* and *FOXO3A* mRNAs tended to be or were significantly higher in the HRT users (*P* = 0.060 and 0.039, respectively), whereas *FOXO1A* mRNA was not significantly modulated (Fig. 2C). Consequently, miR-142-3p was here excluded from the further analyses, as it was not predicted to target other components of the insulin/IGF-1 pathway.

Only limited amount of protein samples from muscle biopsies was available. That was used to study the protein expression of IGF-1R and FOXO3A (Fig. 2D and E). The mean protein level of IGF-1R in the HRT users was 134% of the mean value of their nonusing co-twins (*n* = 9 pairs, *P* = 0.039). Also, the protein level of FOXO3A in the HRT users was 161% of the mean value of the nonusers (*n* = 3 pairs); however, there were samples from only three pairs available which prohibited pair-wise comparisons and statistically testing.

### MiRNA regulation of IGF-1R, FOXO1A, and FOXO3A 3′UTRs

**Figure 3:**
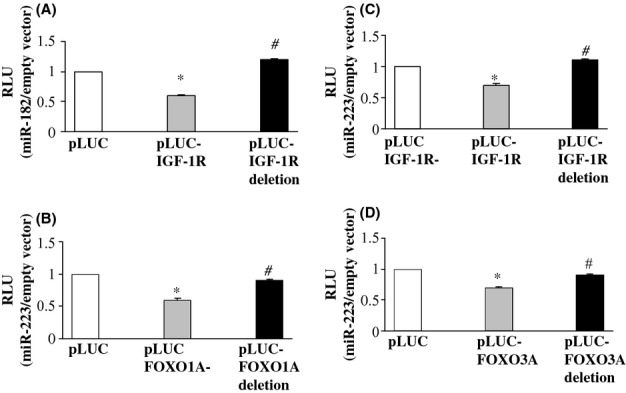
Functional binding assays. (A) Binding of miR-182 to the 3′UTR region of IGF-1R. (B) Binding of miR-223 to the 3′UTR region of FOXO1A. (C) Binding of miR-223 to the 3′UTR region of IGF-1R. (D) Binding of miR-223 to the 3′UTR region of FOXO3A. Each pLUC vector contains the CMV promoter, the firefly luciferase gene, the 3′ UTR of a target gene, and a SV40 terminator sequence. When a miRNA is expressed and binds to the 3′ UTR, it results in repression of luciferase gene expression. We used pLUC plasmids encoding for the 3′-UTR regions of the mRNA targets IGF-1R, FOXO1A, and FOXO3A (pLUC IGF-1R, pLUC FOXO1A, and pLUC FOXO3A) and for the 3′-UTR regions of the same mRNA targets containing deletions (pLUC IGF-1R deletion, pLUC FOXO1A deletion, and pLUC FOXO3A deletion). Each pLUC vector was co-transfected into the HEK293T cells with a plasmid-encoding *Renilla* luciferase along with a plasmid-encoding miR-182 or miR-223 or the empty vector. Also pSUPER-scramble oligos were used for co-transfection into the HEK293T cells. No significant differences were observed when pSUPER-scramble oligos were used instead of empty vector. Firefly luciferase values were normalized to *Renilla* luciferase activity, and the ratio of luciferase activity of each construct in the presence or in the absence of exogenous miR-182/-223 was calculated. Data are reported as relative light units (RLU) and in relation to empty vector. Mean value ± SD of three independent experiments are shown. *P ≤ 0.05 pLUC-target vs. pLUC; #*P* ≤ 0.05 pLUC-target-deletion vs. pLUC. (For details see Experimental procedures).

FOXO3A and FOXO1A have been identified as direct targets for miR-182 (Guttilla & White, 2009; Segura *et al*., 2009). In the present study, we demonstrated, using luciferase assay in HEK-293 cells, that IGF-1R also is a target for miR-182 (Fig. 3A). We also demonstrated here that IGF-1R, FOXO1A, and FOXO3A are direct targets for miR-223 (Fig. 3B, C, D). Hence, the *in silico* prediction made with the SID1.0 software was confirmed.

### MiR-223 and miR-182 modulate IGF-1R, FOXO1A, and FOXO3A protein levels

**Figure 4:**
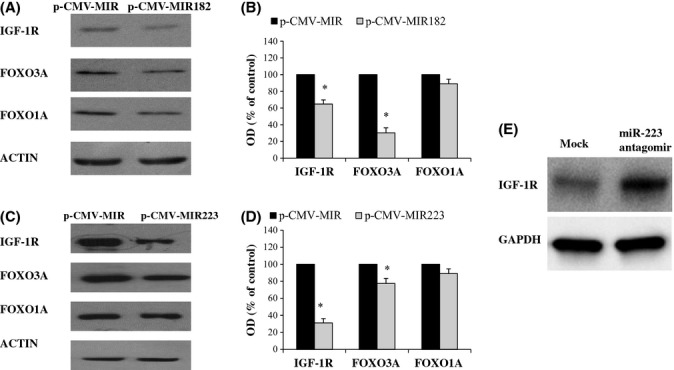
The effects of miR-182 and miR-223 over-expression on protein expression of IGF-1R, FOXO3A, and FOXO1A in MCF-7 cells. (A). Western blot analysis of target proteins in MCF-7 cells transfected with p-CMV-MIR (empty vector) and p-CMV-MIR182. (B) Densitometric quantitation of the blots presented in A. (C) Western blot analysis of target proteins in MCF-7 cells transfected with p-CMV-MIR (empty vector) and p-CMV-MIR223. (D) Densitometric quantitation of the blots presented in C. Results are expressed as percentage of control cells transfected with empty vector (p-CMV-MIR). Data are reported as means ± SD of three independent experiments. OD indicates optical density. *t*-test, *P < 0.05. (E) Western blot analysis of IGF1R protein in MCF-7 cells transfected with mirVana™ miRNA-223 inhibitors (Ambion, USA) hsa-miR-223 ID:MH12301 (miR-223 antagomir), anti-miR negative control #1 (Mock). Results are expressed as percentage of control cells transfected with (anti-miR negative control #1). Data are reported as means ± SD of three independent experiments. OD indicates optical density. *t*-test, *P < 0.05.

Previously, it has been shown that miR-182 over-expression represses FOXO1A and FOXO3A in breast cancer and melanoma cells, respectively (Guttilla & White, 2009; Segura *et al*., 2009). To determine the effects of gain-of-function of miR-182/223, we used MCF-7 cells known to be highly responsive to E_2_. Consequently, MCF-7 cells were transfected with pCMV-MIR182, pCMV-MIR223, and pCMV-MIR (=empty or scrumble vectors) to induce their overexpression and to analyze the protein expression of their targets, that is, IGF-1R, FOXO3A, and FOXO1A. We found that miR-182 over-expression represses IGF-1R and FOXO3A in MCF-7 cells (*P* < 0.05), while the effects on FOXO1A protein expression were milder (Fig. 4A and B). In line with this, transfection-induced over-expression of mature miR-223 significantly inhibited IGF-1R and FOXO3A protein levels (*P* < 0.05), while milder effects on FOXO1A protein expression were observed (Fig. 4C and D). In addition, the loss-of-function of miR-223 resulted in an evident increase in the protein expression of IGF-1R (Fig. 4E).

### Estradiol regulates miR-182 and miR-223 expression and their identified targets leading to activation of insulin/IGF-1 signaling pathway in MCF-7 cells

**Figure 5:**
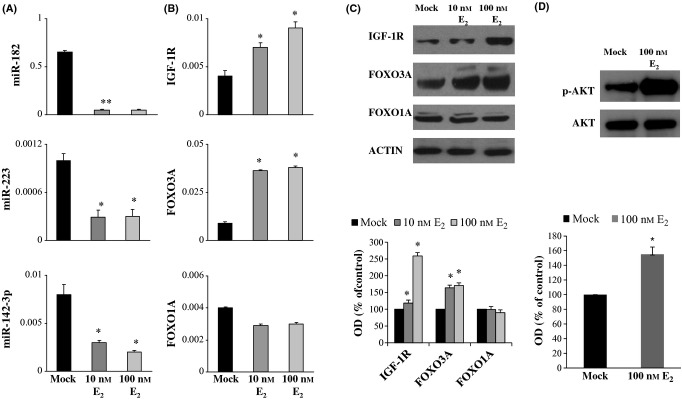
MiR-182, miR-223, and miR-142-3p expression and their target mRNA and protein levels under estrogen stimulation in MCF-7 cells. (A) qPCR analyses of miRNA expression normalized with *RNU44* expression in MCF-7 cells treated for 72 h with 10 nm, 100 nm E2, or mock. (B) qPCR analyses of target mRNAs in MCF-7 cells treated for 72 h with 10 nm, 100 nm E2, or mock. Samples were normalized with GAPDH expression. (C) Western blots and densitometric data of target proteins, normalized with b-actin levels in MCF-7 cells treated for 72 h with 10 nm, 100 nm E2, or mock. (D) Western blot analysis of phosphorylated AKT and total AKT in MCF-7 cell treated with 100 nm estradiol or mock for 24 h. The amount of phosphorylated AKT was analyzed by densitometry and normalized against AKT expression. Data are expressed as percentage of control (mock) and reported as means ± SD of three independent experiments. OD indicates optical density. *t-*test, *P < 0.05.

We used MCF-7 breast cancer cells to show that the identified miRNAs are regulated by E_2_ stimulation. MCF-7 cells are one of the *in vitro* models most widely used to study estrogen signaling, as they express high levels of estrogen receptor alpha (ERα) and are strongly growth-stimulated under exposure to E_2_. In MCF-7 cells, 10 (data not shown) and 100 nm E_2_ treatment induced significant down-regulation of all three miRNAs (*P* < 0.05 for all; Fig. 5A). E_2_ treatment also induced up-regulation of *IGF-1R* and *FOXO3A* mRNA expression (Fig. 5B) as well as up-regulated protein levels (Fig. 5C). Nevertheless, *FOXO1A* was not significantly modulated. A dose-dependent effect was also observed in protein levels (Fig. 5C). Furthermore, Western blot analyses showed E_2_-induced increase in the phosphorylation of AKT in comparison with untreated control cells (Fig. 5D).

### Estradiol suppresses miRNA expression and activates insulin/IGF-1 signaling pathway in human myoblasts and in mouse mature muscle cells

**Figure 6:**
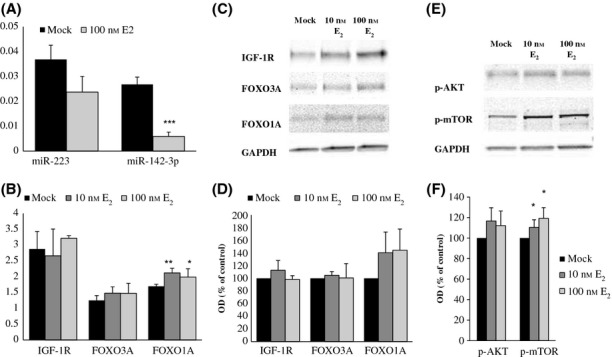
The effects of estrogen stimulation on the expression of miR-182, miR-223, and miR-142-3p and their targets in infant female *quadriceps femoris*-derived myoblasts. (A) Quantitative PCR analyses of miRNA transcripts normalized with *RNU44* in human myoblasts treated for 72 h with 100 nm estradiol or mock. Note: The expression of miR-182 in these myoblasts was too low to be accurately measured. (B) qPCR analyses of target mRNAs (IGF-1, FOXO3A, FOXO1A) in human myoblasts treated with 10 nm and 100 nm estradiol or mock for 72 h. (C) Representative Western blots of target proteins, IGF-1R, FOXO3A and FOXO1A, and GAPDH, in human myoblasts treated with 10 nm and 100 nm estradiol or mock for 72 h. (D) Densitometry data of Western blots normalized with GAPDH. (E) Representative Western blots showing phosphorylation of AKT and mTOR proteins in myoblasts treated with 10 nm and 100 nm estradiol or mock for 72 h. (F) Densitometry data of Western blots normalized with GAPDH. Data are presented as percentage of control (mock) and reported as means ± SD of three independent experiments. OD indicates optical density. *t*-test, ***P < 0.001*,* **P < 0.01, *P < 0.05.

To find out whether E_2_-mediated regulation of the expression of miRNAs and their targets exists specifically in myogenic cells themselves, without other cell types potentially present in muscle biopsies, we exposed human myoblasts derived from *quadriceps femoris* muscle of a newborn girl to E_2_. Both miR-223 and miR-142-3p were down-regulated by 100 nm E_2_, the difference being highly significant for miR-142-3p (Fig. 6A). Concerning the miRNA targets (IGF-1R, FOXO3A and FOXO1A), the expression of FOXO1A increased significantly at mRNA level (Fig. 6B) and tended to increase at protein level (Fig. 6C and D). Furthermore, an increase was found in both the phosphorylation of AKT and mammalian target of rapamycin (mTOR) (Fig. 6E and F) in the human myoblasts following exposure to E_2_. The phosphorylation of mTOR was statistically significantly more abundant in E_2_-exposed muscle cells than in mock-treated cells (*P* < 0.05).

**Figure 7:**
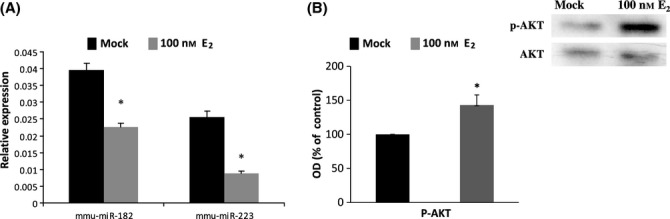
The effects of estrogen stimulation on miRNA transcripts and phosphorylation of AKT protein in young male mouse diaphragm muscle. (A) qPCR analyses of miRNA transcripts normalized to U6B in mouse muscle stripes treated with 100 nm estradiol or mock for 24 h. (B) Western blot analysis of phosphorylated AKT and total AKT in mouse muscles treated with 100 nm estradiol for 24 h. The amount of phosphorylated AKT was analyzed by densitometry and normalized against AKT expression. Data are presented as percentage of control (mock) and reported as means ± SD of three independent experiments. OD indicates optical density. *t*-test, *P < 0.05.

To confirm the ability of E_2_ to activate insulin/IGF-1 signaling pathway in skeletal muscle, we used mouse *diaphragm* muscle cells. We found out that *diaphragm* muscle cells of young male mouse showed down-regulation of both miR-182 and miR-223 under exposure to 100 nm estradiol for 24 h (Fig. 7A) concomitantly with increased AKT phosphorylation (*P* < 0.05) (Fig. 7B).

## Discussion

A co-twin case–control study design with postmenopausal MZ twin sisters discordant for the use of long-term hormone replacement therapy (HRT) was utilized to identify muscular estrogen-sensitive miRNAs and their targets as a mechanism of skeletal muscle regulation by HRT. The most noteworthy novel finding was that in skeletal muscle, HRT use associates with down-regulation of miR-182, miR-223, and miR-142-3p, which modulate the expression of central players in the insulin/IGF-1 pathway, namely IGF-1R and FOXO3A. This is in line with our previous studies showing that postmenopausal HRT induces an up-regulation of genes belonging to the IGF-1 signaling cascade (Pöllänen *et al*., 2010; Ahtiainen *et al*., 2012a) and with other studies showing that miR-182 represses FOXO3A (Guttilla & White, 2009; Segura *et al*., 2009). In addition to repressing FOXO3A, we showed, for the first time, that miR-182 also represses IGF-1R. Furthermore, we confirmed that miR-223 also represses both IGF-1R and FOXO3A. *In vitro* analyses verified that estrogen modulates IGF-1R and FOXO3A expression via miRNA down-regulation as well as affects the activity of the insulin/IGF-1 pathway by enhancing AKT and mTOR phosphorylation both in myoblasts and in mature muscle cells. We suggest that this novel mechanism enables positive estrogen impact on the protein balance and insulin sensitivity in skeletal muscle, thus explaining our previous observations of the favorable effects of HRT on adipokine and glucose profile, mobility, muscle power, and body and muscle composition in postmenopausal women (Ronkainen *et al*., 2009; Ahtiainen *et al*., 2012b).

Although the IGF-1 pathway is one of the main regulatory pathways in skeletal muscle (Velloso, 2008), little is known about how it is affected by estrogen. Instead, studies with breast cancer cell lines have yielded much evidence for this two-way cross talk between estrogen and the IGF-1 pathway (Lee *et al*., 2001). Thus, estradiol is known to potentiate the effect of IGF-1 on IGF-1R signaling (Dupont *et al*., 2000), up-regulate several proteins of the IGF-I pathway (Bernard *et al*., 2006), and activate IGF-IR (Song, 2007). Our recent skeletal muscle studies have shown the estrogen and IGF-1 pathways to be linked, as increased IGF-1 pathway activity was found in the muscle of HRT users but not in nonusers (Pöllänen *et al*., 2010; Ahtiainen *et al*., 2012a). This current study gives further support for estrogen-mediated regulation of IGF-1 signaling.

During recent years, studies related to estrogen-responsive cancers have provided compelling evidence on the regulation via up-regulated miRNAs by estrogen both in humans (Segura *et al*., 2009) and experimental animals (Munagula *et al*. 2013). In skeletal muscle, miRNAs are known to have an important role in regulation, development, and function in health and disease (Guller & Russell, 2010), while their hormonal regulation has remained unknown. In women, several muscular risk factors culminate at menopause, when dramatic changes occur in sex hormone status. Our current study reveals for the first time that two estrogen-sensitive miRNAs regulate genes that are involved in the IGF-1 signaling pathway in human skeletal muscle. First, the down-regulation of miR-182 and miR-223 in the HRT users accorded with the higher mRNA and protein expression of IGF-1R, which in all likelihood leads to higher activity of the following PI3K/Akt-pathway in HRT users compared to nonusers. Binding of IGF-1 to its receptor in skeletal muscle is known to induce a cascade of downstream effects on, for example, proliferation, differentiation, apoptosis, and glucose metabolism (Denley *et al*., 2005). The activation of downstream target PI3K leads to the activation of AKT, which further stimulates protein synthesis via mTOR. Activation of the IGF-1 pathway also inhibits protein degradation via the ubiquitin-proteasome system by phosphorylating and repressing the transcription factors of the FOXO family (Bodine *et al*., 2001). In the current study, we were able to confirm E_2_-mediated increment in the phosphorylation of AKT in mouse muscle and in the phosphorylation of mTOR in human myoblasts.

Furthermore, FOXOs are also required for another proteolytic system, that is, the autophagy lysosome pathway (Mammucari *et al*., 2007) shown to be important for disposal of damaged and toxic proteins and dysfunctional cell organelles. Basal autophagy is highly essential for muscle fiber homeostasis and maintenance of their integrity, while autophagy inhibition enhances muscle loss (Masiero *et al*., 2009). Specifically, the role of FOXO3A as an inducer of autophagy is supported by the finding that transfection of adult muscle fibers with constitutively active FOXO3A causes accumulation of autophagic vacuoles, whereas fasting-induced autophagy is blocked by dominant-negative FOXO3A and by RNA interference-mediated FOXO3A knockdown. Furthermore, FOXO3A is required for the up-regulation of autophagy-related genes such as LC3 and Bnip3 (Mammucari *et al*., 2007). On the other hand, inhibition of autophagy by muscle-specific knockout of the autophagy gene Atg7 was found to cause muscle atrophy accompanied by decrease in muscle force with accumulation of altered mitochondria and aberrant concentric membranous structures. Inhibition of autophagy also exacerbated muscle loss during denervation and fasting suggesting that the persistence of dysfunctional organelles affects the progression of muscle atrophy (Masiero *et al*., 2009). Interestingly, a recent work by Luo *et al*. (2013) demonstrated in aged animal model that resistance exercise training up-regulated the expression of total FOXO3A, among other mediators (Luo *et al*., 2013).These data suggest that the benefits of resistance exercise are associated with increased autophagy activity accompanied by modulation of IGF-1, the AKT/mTOR, and AKT/FOXO3A signaling pathways in aged skeletal muscles (Luo *et al*., 2013). Consequently, it is tempting to suggest that HRT activates in skeletal muscle regulatory routes comparable with those affected by resistance-type physical training.

In addition to protein homeostasis, the IGF-1-pathway plays a central role in muscle metabolism by stimulating glucose transport and glycogen and lipid synthesis following the activation of insulin/IGF-1 receptors (Saltiel & Kahn, 2001). Of the myomiRs, miR-1 and miR-133a have been connected with insulin sensitivity and glucose homeostasis (Granjon *et al*., 2009; Gallagher *et al*., 2010). We found no differences in either of these particular miRNAs between the twin sisters discordant for HRT or in other miRNAs known to be involved in the insulin sensitivity of skeletal muscle (Guay *et al*., 2011). Instead, we found two miRNAs, miR-182 and -223, participating in the main pathway of the body’s glucose homeostasis. Interestingly, the insulin/IGF-1 signaling pathway is an important, evolutionarily conserved pathway influencing aging and longevity. The *FOXO3A* gene in particular has been strongly associated with human longevity and with many phenotypes linked to healthy aging, among which is the preservation of insulin sensitivity (Franceschi *et al*., 2005; Bonafè & Olivieri, 2009). On the strength of these studies, it is possible that miRNA-mediated up-regulation of *FOXO3A* in HRT users has a role in the previously observed signs of better glucose profile, insulin sensitivity, and muscle condition in HRT users, but not in nonusers (Ronkainen *et al*., 2009; Ahtiainen *et al*., 2012b).

Age-related muscle wasting and, subsequently, the development of sarcopenia are influenced by gradually declining physical activity and by several biological processes, such as hypogonadic hormonal changes, fat infiltration, and insulin resistance. Aging as such is associated with decreased circulating IGF-1 levels, decreased IGF-1R content and IGF-1R phosphorylation in muscle, and dysfunction of the postreceptor signaling pathways (Barbieri *et al*., 2003). Furthermore, the skeletal muscle anabolic response to IGF-1 administration is weakened in the elderly (Cao *et al*., 2007). Rightly, modulation of the IGF system is suggested as a reasonable strategy to improve muscle condition in older adults. In the present study, we confirmed that estrogen-regulated miRNAs, that is, miR-182, miR-223 and miR-142-3p, exist in skeletal muscle of postmenopausal women. Specifically, miR-182 and miR-223 participate in the modulation of the insulin/IGF-1 pathway signaling. We suggest that the observed miRNA-mediated enhancement of the target genes’ *IGF-1R* and *FOXO3A* expression as well as activation of IGF-1 pathway signaling via phosphorylation of AKT and mTOR is an important mechanism for positive estrogen impact on skeletal muscle in postmenopausal women using hormone replacement therapy.

## Experimental procedures

### Study design and participants

This study is a part of a larger research project, ‘Sarcopenia and Skeletal Muscle Adaptation to Postmenopausal Hypogonadism: Effects of Physical Activity and Hormone Replacement Therapy in Older Women – a Genetic and Molecular Biology Study on Physical Activity and Estrogen-related Pathways’ (SAWEs), designed to investigate the molecular events involved in maintaining proper muscle mass and functions postmenopause (Ronkainen *et al*., 2009). The participants for this study were recruited from the Finnish Twin Cohort (*n* = 13 888 pairs) (Kaprio & Koskenvuo, 2002). An invitation was sent to all MZ female twin pairs born in 1943–1952 (*n* = 537 pairs). Only twin pairs in which one co-twin was a current HRT user and the other co-twin was not currently using HRT were invited to respond to the invitation. Of all the responders (*n* = 114 pairs), twin pairs where one sister had never used HRT, while the other sister was a current user, and who reported willingness to participate in the laboratory measurements, were contacted (*n* = 21 pairs). Finally, a total of 16 MZ pairs aged 54–62 years discordant for HRT use and without any predefined contraindications participated in the study, as described previously (Ronkainen *et al*., 2009). One twin pair turned out to be dizygotic and was excluded from the further analyses. Consequently, 15 pairs of HRT discordant MZ twin pairs aged 57.8 ± 2.0 years were finally included to the study group. The HRT users consisted of five women using estradiol-only preparations (1–2 mg), six women on combined treatment including estrogenic (1–2 mg) and progestogenic compounds, and four women using tibolone (2.5 mg). In the present study, our focus was on estrogen deprivation and replacement (estradiol-only plus combined treatments). Therefore, tibolone users were excluded from the study. The mean duration of HRT usage was 7.5 ± 3.9 years (range 2–16 years) in twin pairs from whom muscle samples were available.

### Estradiol measurement

Serum E_2_ levels were assessed in duplicate by extraction RIA qualified for low serum steroid levels, as described previously (Ankarberg-Lindgren & Norjavaara, 2008).

### Body and thigh muscle composition

Body mass index (BMI) was calculated from height and weight measured with standard procedures. Percentage body fat was measured with bioelectrical impedance [InBody (720), Biospace Co. Ltd., Seoul, Korea]. Computed tomography (CT) scans (Siemens Somatom Emotion scanner, Siemens AG, Erlangen, Germany) of the thigh were obtained from the midpoint between the greater trochanter and the lateral joint line of the knee. The scans were analyzed using software developed at the University of Jyväskylä (Jyväskylä, Finland) for cross-sectional CT image analysis (Geanie 2.1, Commit Ltd, Espoo, Finland). The software separates fat and lean tissue based on given radiological density limits. Total thigh muscle and fat cross-sectional areas (CSA) were analyzed, and the relative proportion of muscle in the CSA of the whole thigh was calculated. Muscle power, that is, the ability of the neuromuscular system to produce the greatest possible force as rapidly as possible, was assessed as vertical jumping height on a contact mat, as described previously (Ronkainen *et al*., 2009).

### Muscle tissue sampling and myoblast cell culture

Muscle (*m. vastus lateralis*) needle biopsies from the study subjects were taken under standard fasted conditions under local anesthesia with supine participants. Tissue samples were snap frozen and stored at −80 °C for further analyses.

Primary human muscle cell line (myoblasts) obtained from *quadriceps femoris* muscle biopsy of a 5-day-old, healthy female infant was kindly provided by Dr Gillian Butler-Browne and Dr Vincent Mouly. Myoblast cells were maintained and cultivated in phenol-free DMEM supplemented with 20% 199 medium, 20% fetal bovine serum, and 50 μg mL^−1^ gentamycin (all from Life Technologies, Carlsbad, CA, USA).

*Diaphragm* muscles from 2-month-old male mice were excised and divided into stripes of approximately 10 mg. Stripes of muscles were placed onto culture plates in serum-free DMEM so that the muscle piece was covered by the medium.

### MiRNA profiling

Mature miRNA profiling was performed using an Applied Biosystems 7900 HT real-time PCR instrument and human MicroRNA Array pool A (Applied Biosystems, Foster City, CA, USA), containing 365 different, most common human miRNA assays in addition to selected small nucleolar RNAs (snoRNAs). RNA was converted to cDNA by priming with a mixture of looped primers (MegaPlex kit, Applied Biosystems) in compliance with the manufacturer’s instructions. Preamplification was performed using 3 μL of input RNA with PreAmp kit (Applied Biosystems). 9 μL of preamplified cDNA was used for RT–PCR with MicroRNA Array pool A.

### qPCR validation

Real-time quantitation to measure miRNA expression was performed with the TaqMan miRNA reverse transcription kit and miRNA assay (Applied Biosystems) with some modifications. Briefly, total RNA was reverse transcribed (RT) by a TaqMan MicroRNA RT kit. 5 μL of RT reactions contained 1 μL of each miR-specific stem-loop primer, 1.67 μL of input RNA, 0.4 μL of 10 mm dNTPs, 0.3 μL of reverse transcriptase, 0.5 μL of 10 × buffer, 0.6 μL of RNAse inhibitor diluted 1:10, and 0.5 μL of H_2_O_2_. The mixture was incubated at 16 °C for 30 min, at 42 °C for 30 min, and at 85 °C for 5 min. Subsequently, quantitative real-time PCR was performed. For 5 μL of PCR reaction, 0.25 μL of 20x TaqMan MicroRNA Assay containing PCR primers and probes (5′-FAM), 2.75 μL of 2x TaqMan Universal Master mix, no UNG (Applied Biosystems), and 2.25 μL of RT product was mixed. The reaction was first incubated at 95 °C for 2 min followed by 40 cycles of 95 °C for 15 min and 60 °C for 1 min. Data were analyzed with real-time PCR Opticon Monitor version 2 (MJ Research, Waltham, MA, USA), with the automatic Ct (cycle threshold) setting for adapting baseline and threshold for Ct determination. MiRNA fold changes between different groups were calculated by the delta Ct method. U44 small nucleolar RNA (RNU44) was used as the housekeeping small RNA reference gene. Each reaction was performed in duplicate.

### Putative targets identification

To increase speed and accuracy of the identification of putative mRNA targets common to more than one miR, a computer program, named SID1.0 (simple String IDentifier), previously developed by our group, was used (Albertini *et al*., 2011). This program is based on an exhaustive search strategy and is specifically designed to screen shared data (target genes, miRs and pathways) available from the PicTar and DIANA-MicroT 3.0 databases. Common pathways of specific miRs were identified using the DIANA-microT 3.0 target prediction program (http://diana.cslab.ece.ntua.gr/microT/).

### Gene expression analysis

Total RNA from muscle tissue samples, myoblasts, and MCF-7 cells was extracted using Trizol reagent (Invitrogen, Carlsbad, CA, USA). For the qPCR analysis, one microgram of RNA was reverse transcribed into cDNA (TaqMan Reverse Transcription Reagents, N808-0234, Applied Biosystems). TaqMan Gene Expression Assays (Applied Biosystems) were used to investigate the expression of IGF-1R (Hs99999020_m1), FOXO1A (Hs01054576_m1), and FOXO3A (Hs00818121_m1). Assays were performed with an Applied Biosystems ABI 7300 unit using standard PCR conditions as recommended by the manufacturer. Samples were run in triplicate, and the reference sample was included in all plates to control for interassay variation. GAPDH (Hs99999905_m1) was used for endogenous control.

### Western immunoblot analyses

For human muscle samples, total proteins were extracted using tissue extraction reagent I-buffer (Invitrogen) supplemented with protease and phosphatase inhibitor cocktail (Thermo Scientific, Rockford, IL, USA). Total proteins from cell experiments and mouse muscle were extracted using RIPA buffer (150 mm NaCl, 10 mm Tris, pH 7.2, 0.1% SDS, 1.0% Triton X-100, 5 mm EDTA, pH 8.0) containing protease inhibitor cocktail (Roche Applied Science, IN, USA). Protein concentration was determined using Pierce BCA-assay for human muscle samples and myoblasts and Bradford Reagent (Sigma-Aldrich, St Louis, MO, USA) for other experiments. Total protein extracts (40 μg) were separated by Criterion TGX precast gels 4–20% (human muscle/myoblasts) or by 10% SDS-PAGE (MCF-7 cells/mouse muscle) and transferred to nitrocellulose or PVDF membrane (Whatman, Dassel, Germany). For human muscle and myoblast Western blots, the membranes were blocked in Odyssey blocking buffer (LI-COR, Licoln, NE, USA) and washed in PBS with 0.1% Tween-20 (PBS-T) before incubation with primary antibodies against FOXO3A, IGF-1R, p-AKT, or p-mTOR diluted 1:1000 (Cell Signalling Technology, Beverly, MA, USA). GAPDH antibody diluted 1:40000 was used to control loading and quantitation (Sigma-Aldrich, St Louis, MO, USA). IRDye 800CW donkey anti-rabbit IgG (LI-COR) was used as secondary antibody, and proteins were visualized and quantified using Odyssey CLx (LI-COR). For the MCF-7 cell and mouse muscle experiments, membranes were blocked in TBS with 0.1% Tween-20 (TBS-T) containing 5% fat-free dry milk for 60 min and then incubated overnight at 4 °C with rabbit polyclonal primary antibodies against FOXO1A, FOXO3A, IGF-1R, p-AKT, and total AKT diluted 1:1000 (Cell Signalling Technology) and against β-actin diluted 1:10000 (Santa Cruz, CA), which was used to check the uniformity of blotting. Membranes were washed in TBS-T and incubated 60 min with secondary antibody diluted 1:10000 (Sigma-Aldrich) followed by washing in TBS-T. Proteins were visualized by ECL as instructed by the manufacturer (Amersham, Piscataway, NJ, USA) and quantified using Quantity One software (Bio-Rad Laboratories, Hercules, CA, USA).

### Experiments for phosphorylation of AKT and mTOR

Myoblasts were treated for 72 h with 10 nm E_2_, 100 nm E_2_, or solvent alone and analyzed in Western blots to detect phosphorylation of AKT (SER 473, Cell Signalling #9271) and mTOR (SER 2448, Cell Signalling #2971). Phosphorylation was visualized and quantified using Odyssey CLx (LI-COR). GAPDH (Sigma-Aldrich) was used to standardize loading.

Stripes of mouse diaphragms were exposed either to 100 nm E_2_ or solvent alone for 24 h. Stripes were homogenized and lysed in RIPA buffer containing phosphatase and proteases inhibitors (mini cOmplete and PhosSTOP, Roche) and analyzed by Western blots to detect SER 473 phosphorylation of AKT. Image-J was used to compute densitometry of bands. Total AKT (Cell Signalling #9272) was used to standardize loading.

### Binding analyses (Luciferase Activity assay)

The rationale of this assay is based on the translational inhibition of the firefly luciferase gene by cloning of miRNA-targeted sequences in its 3′UTR (3′untranslated regions). P-CMV-MIR182, p-CMV-MIR223, and p-CMV-MIR (empty vector) plasmids were shipped from OriGene (OriGene Technologies Inc., Rockville, MD, USA). The plasmids used in the luciferase assays were generated by cloning oligonucleotides bearing wild-type (wt) or deleted (del) miR-182/223 target pairing site of *IGF-1R*, *FOXO1A,* and *FOXO3A* genes downstream of the stop codon in pMIR-REPORT-Luciferase (pLUC, Ambion Inc., Austin, Texas, USA), between the SpeI and HindIII restriction sites. The sequences of the oligonucleotides (5′-3′) used for the luciferase activity assays are presented in Table S2. HEK-293 cells were transfected with 100 ng of pLUC, pLUC-182/223-target or pLUC-182/223-target-del, 0.9 μg of pCMV-MIR182/223 and pCMV-MIR, and 50 ng of pRL-null renilla luciferase. Cellular extracts were tested with the Dual Luciferase Assay (Promega Corp, Madison, WI, USA), according to the manufacturer’s instructions, 48 h after transfection, using a Synergy HT luminometer (BioTek Instruments Inc, Winooski, VT, USA). Values were normalized according to renilla luciferase activity, and the ratio of firefly luciferase activity of each construct was calculated either in the presence or in the absence of exogenous miR-182/223.

### Functional analyses

MCF-7 cells (human breast adenocarcinoma cell line) were used for the functional assay. MCF-7 cells are one of the *in vitro* models most widely used to study E_2_-signaling, as they express high levels of ERα and are strongly growth-stimulated by E_2_. MCF-7 cells were maintained in Dulbecco’s modified Eagle’s medium (DMEM, Euroclone, Milano, Italy) supplemented with 10% fetal bovine serum (FBS), 1% penicillin/streptomycin, and 1% L-glutamine (Euroclone). Three days before the experiments, the cells were switched to phenol red-free DMEM (Sigma-Aldrich) containing 10% charcoal/dextran-stripped FBS (Sigma-Aldrich), 1% penicillin/streptomycin, and 1% L-glutamine (Euroclone). Where indicated, treatments included vehicle control (100% EtOH) and 17β-estradiol (10 or 100 nm; Sigma-Aldrich) for 72 h. Transient transfection of miRs was performed with FuGENE transfection reagent (Roche Applied Science, Indianapolis, USA), according to the manufacturer’s instructions. In brief, 8 × 10^4^ cells were plated in 6-well plates and kept overnight for attachment. The next day, the cells were transfected with pCMV-MIR182/223 and pCMV-MIR (OriGene) or mirVana™ miRNA inhibitors (Ambion, USA) hsa-miR-223 ID:MH12301 (miR-223 antagomir), anti-miR negative control #1 (Mock) (Fig 3). The FuGENE transfection method was optimized by testing different quantities of reagent and miR; FuGENE (μL)/miR (μg) ratios of 3:1 in particular were found to be optimal. Hormonal treatments were initiated 48 h after transfection. The medium was changed 72 h after transfection with fresh phenol red-free DMEM supplemented with 0.5% charcoal/dextran-stripped FBS.

### Statistical analysis

MiRNAs expressed at a detectable level in more than 80% of samples in the microRNA array were included in the final data analysis. As a normalization correction factor, the miRNAs were compared based on their expression relative to the overall miRNA expression on each array, using median normalization analysis (ΔCt). MiRNAs showing a difference in mean ΔCt higher than 1.5 (ΔΔCt > 1.5 and ΔΔCt < −1.5) between the studied groups were selected. Fold change was calculated based on the estimated mean difference (2^(-ΔΔCT)). The statistical analyses for the group comparisons included either paired-samples t-test or nonparametric Wilcoxon signed ranks tests, depending on the normal distribution of the means tested by the Shapiro–Wilk-test. *P* values less than 0.05 were considered significant.

### Ethics

The study follows the guidelines of good clinical and scientific practice as well as the Helsinki Declaration. The ethics committee of the Central Finland Health Care District approved the SAWEs study on June 6, 2006. A written informed consent form explaining the possible risks and personal benefits associated with the examinations and permission for their data to be used for research purposes only and in publications was signed by the participants before the measurements.

## Supporting Information

**Table S1** Common targets for hsa-miR-142-3p (250 elements), hsa-miR-182 (841 elements) and hsa-miR-223 (202 elements). **Table S2** The oligonucleotides used for the luciferase activity assays.
