# Supplementary material for: Hormone replacement therapy enhances IGF-1 signaling in skeletal muscle by diminishing miR-182 and miR-223 expressions: a study on postmenopausal monozygotic twin pairs
Source: Aging Cell. 2014 Jul 18;13(5):850–61. doi: 10.1111/acel.12245 (PMC4331762; doi:10.1111/acel.12245)
Supplement: Supplementary file 1 — Table S1 Common targets for hsa-miR-142-3p (250 elements), hsa-miR-182 (841 elements) and hsa-miR-223 (202 elements). Table S2 The oligonucleotides used for the luciferase activity assays. [file acel0013-0850-sd1.docx]

**Table S1**. Common targets for hsa-miR-142-3p (250 elements), hsa-miR-182 (841 elements) and hsa-miR-223 (202 elements). Three targets are common to all the miRs used in the analysis (FOXO1A; SOX11 and ZCCH14), while IGF-1R and FOXO3A are common to miR-182 and miR-223. SID1.0 software and the TargetScan 5.2 database were used for this analysis. Bold letters highlight the predicted common targets of selected miRs, belonging to the IGF-1 pathway; See also the Venn diagram in Figure 2.

| **Target gene** | **Gene name** | **Common miRNAs** |
| --- | --- | --- |
| **FOXO1A**  SOX11  ZCCH14 | **forkhead box O1**  SRY (sex determining region Y)-box 11  zinc finger, CCHC domain containing 14 | hsa-miR-142-3p, hsa-miR-182 and hsa-miR-223 |
| SLC37A3  APC  EML4  MYH10  ZNF395  ACVR2A  PURB  EPAS1  RASA1  ABHD13  **FOXO3A**  TMEM47  SP3  **IGF1R**  FBXW7  EBF3  MEF2C  UBE2W  MGC24039  ELL2  PRKCE  SR140  SLC39A1  MEF2D  BRPF3  ARMC1  FRMD4A  BRWD1  KPNA1  BAI3  FAM120C  PDE4D  PHF20L1  NFASC  SCN1A  SCN2A  SCN3A | solute carrier family 37 (glycerol-3-phosphate transporter), member 3  adenomatous polyposis coli  echinoderm microtubule associated protein like 4  myosin, heavy chain 10, non-muscle  zinc finger protein 395  activin A receptor, type IIA  purine-rich element binding protein B  endothelial PAS domain protein 1  RAS p21 protein activator (GTPase activating protein) 1  abhydrolase domain containing 13  **forkhead box O3**  transmembrane protein 47  Sp3 transcription factor  **insulin-like growth factor 1 receptor**  F-box and WD repeat domain containing 7  early B-cell factor 3  myocyte enhancer factor 2C  ubiquitin-conjugating enzyme E2W (putative)  hypothetical protein MGC24039  elongation factor, RNA polymerase II, 2  protein kinase C, epsilon  U2-associated SR140 protein  solute carrier family 39 (zinc transporter), member 1  myocyte enhancer factor 2D  bromodomain and PHD finger containing, 3  armadillo repeat containing 1  FERM domain containing 4°  bromodomain and WD repeat domain containing 1  karyopherin alpha 1 (importin alpha 5)  brain-specific angiogenesis inhibitor 3  family with sequence similarity 120C  phosphodiesterase 4D, cAMP-specific (phosphodiesterase E3 dunce homolog, Drosophila)  PHD finger protein 20-like 1  neurofascin homolog (chicken)  sodium channel, voltage-gated, type I, alpha subunit  sodium channel, voltage-gated, type II, alpha subunit  sodium channel, voltage-gated, type III, alpha subunit | hsa-miR-182 and hsa-miR-223 |
| USP6NL  C20orf194  ITGB8  RICTOR  ZBTB41  RAC1  ZNF831  DCUN1D4  ANK3  SNF1LK  MARCKS  MORF4L2  TAOK1  GNAQ  SAMD12  BNC2  TBL1X  TEX2  TEAD1  CCNJ  SYPL1  CTTN  BACH2  EDEM3  VAMP3  RARG  CLCN5  ATG16L1  JMJD1C  GPR85  TET2  TP53INP2  PPP3R1  PPP3CA  PCGF3  MLL  ARHGEF12  UNKL  KLF13  C16orf70  TMEM115  ABL2 | USP6 N-terminal like  chromosome 20 open reading frame 194  integrin, beta 8  rapamycin-insensitive companion of mTOR  zinc finger and BTB domain containing 41  ras-related C3 botulinum toxin substrate 1 (rho family, small GTP binding protein Rac1)  zinc finger protein 831  DCN1, defective in cullin neddylation 1, domain containing 4 (S. cerevisiae)  ankyrin 3, node of Ranvier (ankyrin G)  SNF1-like kinase  myristoylated alanine-rich protein kinase C substrate  mortality factor 4 like 2  TAO kinase 1  guanine nucleotide binding protein (G protein), q polypeptide  sterile alpha motif domain containing 12  basonuclin 2  transducin (beta)-like 1X-linked  testis expressed 2  TEA domain family member 1 (SV40 transcriptional enhancer factor)  cyclin J  synaptophysin-like 1  cortactin  BTB and CNC homology 1, basic leucine zipper transcription factor 2  ER degradation enhancer, mannosidase alpha-like 3  vesicle-associated membrane protein 3 (cellubrevin)  retinoic acid receptor, gamma  chloride channel 5 (nephrolithiasis 2, X-linked, Dent disease)  ATG16 autophagy related 16-like 1 (S. cerevisiae)  ATG16 autophagy related 16-like 1 (S. cerevisiae)  G protein-coupled receptor 85  tet oncogene family member 2  tumor protein p53 inducible nuclear protein 2  protein phosphatase 3 (formerly 2B), regulatory subunit B, alpha isoform  protein phosphatase 3 (formerly 2B), catalytic subunit, alpha isoform  polycomb group ring finger 3  myeloid/lymphoid or mixed-lineage leukemia (trithorax homolog, Drosophila)  Rho guanine nucleotide exchange factor (GEF) 12  unkempt homolog (Drosophila)-like  Kruppel-like factor 13  chromosome 16 open reading frame 70  transmembrane protein 115  v-abl Abelson murine leukemia viral oncogene homolog 2 (arg, Abelson-related gene) | hsa-miR-182 and hsa-miR-142-3p |
| SLC37A3  APC  EML4  MYH10  ZNF395  ACVR2A  PURB | solute carrier family 37 (glycerol-3-phosphate transporter), member 3  adenomatous polyposis coli  echinoderm microtubule associated protein like 4  myosin, heavy chain 10, non-muscle  zinc finger protein 395  activin A receptor, type IIA  purine-rich element binding protein B | hsa-miR-142-3p and hsa-miR-223 |

**Table S2.** The oligonucleotides used for the luciferase activity assays.

| **Oligonucleotide name** | **Sequence (5’-3’)** |
| --- | --- |
| *miR-182/IGF-1R luciferase activity assay:* | |
| IGF-1R_forward | ctagtcattttaacgctgcctaattttgccaaaatcctgaactttctccctcaa |
| IGF-1R_reverse | AGCTTTGAGGGAGAAAGTTCAGGATTTTGGCAAAATTAGGCAGCGTTAAAATGA |
| IGF-1R_del_forward | Ctagtcattttaacgctgcctaattatcctgaactttctccctcaa |
| IGF-1R_del_reverse | AGCTTTGAGGGAGAAAGTTCAGGATAATTAGGCAGCGTTAAAATGA |
| *miR-223/IGF-1R luciferase activity assay:* | |
| IGF-1R_forward | CTAGTTATTCCCTGCCCAAACCCTTAACTGACATGGGCCTTTAAGAACCTTAAA |
| IGF-1R_reverse | AGCTTTTAAGGTTCTTAAAGGCCCATGTCAGTTAAGGGTTTGGGCAGGGAATAA |
| IGF-1R_del_forward | CTAGTTATTCCCTGCCCAAACCCTTTGGGCCTTTAAGAACCTTAAA |
| IGF-1R_del_reverse | AGCTTTTAAGGTTCTTAAAGGCCCAAAGGGTTTGGGCAGGGAATAA |
| *miR-223/FOXO1A luciferase activity assay:* | |
| FOXO1A _forward | CTAGTATTACTTTCCAATTACCTGTAACTGACAGACCAAATTAATTGGCTTTGA |
| FOXO1A _reverse | AGCTTCAAAGCCAATTAATTTGGTCTGTCAGTTACAGGTAATTGGAAAGTAATA |
| FOXO1A _del_forward | CTAGTATTACTTTCCAATTACCTGTGACCAAATTAATTGGCTTTGA |
| FOXO1A _del_reverse | AGCTTCAAAGCCAATTAATTTGGTCACAGGTAATTGGAAAGTAAT |
| *miR-223/FOXO3A luciferase activity assay:* | |
| FOXO3A _forward | CTAGTGTGGGCAAAGCAGACCCTCAAACTGACACAAGACCTACAGAGAAAACCA |
| FOXO3A _reverse | AGCTTGGTTTTCTCTGTAGGTCTTGTGTCAGTTTGAGGGTCTGCTTTGCCCACA |
| FOXO3A _del_forward | CTAGTGTGGGCAAAGCAGACCCTCACAAGACCTACAGAGAAAACCA |
| FOXO3A _del_reverse | AGCTTGGTTTTCTCTGTAGGTCTTGTGAGGGTCTGCTTTGCCCACA |
